# A dose escalation/expansion study evaluating dose, safety, and efficacy of the novel tyrosine kinase inhibitor surufatinib, which inhibits VEGFR 1, 2, & 3, FGFR 1, and CSF1R, in US patients with neuroendocrine tumors

**DOI:** 10.1007/s10637-023-01359-2

**Published:** 2023-04-19

**Authors:** Arvind Dasari, Erika P. Hamilton, Gerald S. Falchook, Judy S. Wang, Daneng Li, Max W. Sung, Caly Chien, Shivani Nanda, Christopher Tucci, Marjo Hahka-Kemppinen, Andrew Scott Paulson

**Affiliations:** 1 MD Anderson Cancer Center, Houston, TX USA; 2 Sarah Cannon Research Institute/Tennessee Oncology, Nashville, TN USA; 3 Sarah Cannon Research Institute at HealthONE, Denver, CO USA; 4 Florida Cancer Specialists/Sarah Cannon Research Institute, Sarasota, FL USA; 5 City of Hope Comprehensive Cancer Center and Beckman Research Institute, Duarte, CA USA; 6 Tisch Institute at The Icahn School of Medicine at Mount Sinai, New York, NY USA; 7 HUTCHMED International Corporation, Florham Park, NJ USA; 8 Baylor Sammons Cancer Center, Dallas, TX USA

**Keywords:** Surufatinib, Pancreatic neuroendocrine tumors, Extrapancreatic neuroendocrine tumors, Vascular endothelial growth factor receptor

## Abstract

Surufatinib, is a potent inhibitor of vascular endothelial growth factor receptors 1–3; fibroblast growth factor receptor-1; colony-stimulating factor 1 receptor. This Phase 1/1b escalation/expansion study in US patients with solid tumors evaluated 5 once daily (QD) surufatinib doses (3 + 3 design) to identify maximum tolerated dose (MTD), recommended Phase 2 dose (RP2D), and evaluate safety and efficacy at the RP2D in 4 disease-specific expansion cohorts including pancreatic neuroendocrine tumors [pNET] and extrapancreatic NETs [epNET]. MTD and RP2D were 300 mg QD (escalation [n = 35]); 5 patients (15.6%) (Dose Limiting Toxicity [DLT] Evaluable Set [n = 32]) had DLTs. Pharmacokinetics were dose proportional. Estimated progression-free survival (PFS) rates at 11 months were 57.4% (95% confidence interval [CI]: 28.7, 78.2) and 51.1% (95% CI: 12.8, 80.3) for pNET and epNET expansion cohorts, respectively. Median PFS was 15.2 (95% CI: 5.2, not evaluable) and 11.5 (95% CI: 6.5,11.5) months. Response rates were 18.8% and 6.3%. The most frequent treatment-emergent adverse events (both cohorts) were fatigue (46.9%), hypertension (43.8%), proteinuria (37.5%), diarrhea (34.4%). Pharmacokinetics, safety, and antitumor efficacy of 300 mg QD oral surufatinib in US patients with pNETs and epNETs are consistent with previously reported studies in China and may support applicability of earlier surufatinib studies in US patients. **Clinical trial registration**: Clinicaltrials.gov NCT02549937.

**Supplementary Information:**

The online version contains supplementary material available at 10.1007/s10637-023-01359-2.

## Introduction

Neuroendocrine tumors (NETs) are rare, heterogeneous tumors with diverse pathology that arise from secretory neuroendocrine cells originating from multiple organ sites [1]. NETs are heavily vascularized, and express many pro-angiogenic molecules, including vascular endothelial growth factor (VEGF), fibroblast growth factor (FGF), and platelet-derived growth factor [2]. The incidence and prevalence of NETs is increasing steadily across the globe, with the greatest increase in North America [3, 4]. In the US, the yearly incidence between 1973 and 2012 increased from 1.09 to 6.98 per 100,000 [3]. Recommended treatments beyond the use of somatostatin analogs (SSAs) and chemotherapy remain limited for most NET subtypes [5, 6]. Patients with advanced NETs typically have a poor prognosis, with a median overall survival of about ≤ 5 years depending on the stage [7].

Surufatinib is a novel, small molecule, oral, targeted inhibitor of tyrosine kinase VEGF receptors 1, 2, and 3; FGF receptor 1; and colony-stimulating factor 1 receptor. It has been approved in China for patients with advanced pancreatic neuroendocrine tumors (pNET) and extrapancreatic neuroendocrine tumors (epNET). In 2, randomized, Phase 3, registrational studies in pNETs and epNETs conducted in China, surufatinib consistently demonstrated improved efficacy over placebo with an acceptable safety profile [8, 9].

This Phase 1/1b, dose escalation/expansion study (NCT02549937) was initiated in US patients to confirm surufatinib safety and efficacy results in clinical studies performed in China. Here, we report the pharmacokinetics (PK) for all patients in dose escalation/expansion, and the safety and antitumor efficacy for patients in dose escalation and the pNET and epNET dose expansion cohorts.

## Materials and methods

This was an open-label dose escalation and dose expansion surufatinib study in US patients with solid tumors. The primary objective of dose escalation was to evaluate surufatinib safety and tolerability in patients with advanced solid tumors of any type and to determine the maximum tolerated dose (MTD) and/or recommended Phase 2 dose (RP2D) as measured by the incidence of dose-limiting toxicities (DLTs) and adverse events (AEs). Secondary objectives were to evaluate multiple dose surufatinib PK, and antitumor activity in patients with solid tumors according to Response Evaluation Criteria in Solid Tumors (RECIST) version (v) 1.1. The primary objective of dose expansion was to evaluate surufatinib antitumor activity in 4 cohorts, including patients with advanced or metastatic biliary tract cancer, pNETs, epNETs, and soft tissue sarcomas, at the RP2D established during dose escalation. Here, we report data for pNET and epNET expansion cohorts.

### Patients

Eligible patients were ≥ 18 years of age, and in dose escalation had histologically or cytologically documented, locally advanced, or metastatic solid tumors that had progressed on available standard systemic therapy, and for which there was no effective therapy or standard of care. For the dose expansion pNET cohort, patients had low-to-intermediate grade (grade 1 or 2), well-differentiated, unresectable, or metastatic pNETs that had progressed on everolimus, sunitinib, or both. For the dose expansion epNET cohort, patients had low-to-intermediate grade (grade 1 or 2), well-differentiated, unresectable, or metastatic epNETs that had progressed on everolimus. Patients with high-grade (grade 3) NETs, even if well-differentiated, and patients with squamous non-small cell lung cancer were excluded. Patients with functional advanced NETs could have received SSAs for control of secretory symptoms and may have continued to take SSAs during the study, provided that a stable dose had been established over the 2 months prior to enrollment and that the treatment was for control of secretory symptoms only. Some patients with NETs received SSAs in the absence of secretory symptoms with the aim of delaying disease progression. In order to be eligible for the study, these patients were also treated with targeted therapy (sunitinib or everolimus). In these patients, both the targeted therapy and the SSA were discontinued ≥ 4 weeks before the first dose of study drug.

### Study design

#### Dose escalation

Five once daily (QD) surufatinib dose levels were evaluated in dose escalation (50, 100, 200, 300, and 400 mg). A 3 + 3 design was used with a minimum of 3 patients enrolled and observed for toxicity at each dose. Enrollment in the next higher dose proceeded if the 3 patients initially enrolled in a dose cohort completed the DLT assessment window (Cycle [C] 1 Day [D] 1 to C1D28) without experiencing a DLT. If 1 of the initial 3 patients experienced a DLT during the DLT assessment window, additional patients were enrolled at that dose for a minimum of 6 DLT-evaluable patients. If ≤ 1 of the 6 evaluable patients experienced a DLT, dose escalation proceeded to the next predefined dose. If ≥ 2 DLTs were observed in the 6 evaluable patients at a given dose, dose escalation was halted. If this dose was ≥ 50% higher than the previous dose, an intermediate dose was evaluated for toxicity. If the dose was < 50% higher than the previous dose, additional patients were enrolled at the previous dose, if necessary, for a minimum of 6 evaluable patients.

DLT-evaluable patients had not received any preventive treatment during the DLT period, completed the first 28-day treatment cycle with complete safety evaluations, and received ≥ 75% of the assigned surufatinib dose or had a confirmed DLT during the first 28-day treatment cycle. Patients who completed the DLT observation window and were deemed (in the Investigator’s judgment) to be benefiting from surufatinib treatment were permitted to continue surufatinib treatment until disease progression, death, intolerable toxicity, pregnancy, or loss of benefit. Patients with acceptable toxicity and ongoing clinical benefit were permitted to receive surufatinib for up to 1 year at the Investigator’s discretion and with the Sponsor’s agreement.

#### Dose expansion

The expansion phase of the study evaluated the antitumor activity of surufatinib and the safety and tolerability of surufatinib in 4 tumor-specific cohorts at the RP2D determined at the end of dose escalation. Here, we report the safety and antitumor efficacy in the pNET and epNET cohorts.

### Pharmacokinetic evaluations

Blood samples for surufatinib plasma concentrations were collected at pre-dose and 1, 2, 4, 6, 8 and 24 h after dosing on C1D1, C1D15, and C2D1. The 24-hour samples were collected prior to the next dose. Samples were analyzed using a validated, specific, and sensitive liquid chromatography with tandem mass spectrometry assay method. Systemic surufatinib exposure was evaluated based on area under the plasma concentration-time curve (AUC) from 0 to 24 h (AUC_0− 24_); AUC over the dosing interval (AUC_0 − tau_); maximum plasma concentration (C_max_); minimum plasma concentration at steady state (C_min_); time to C_max_ (T_max_); apparent clearance at steady state (CL_ss_/F); and accumulation ratio based on AUC. PK parameters were determined by non-compartmental analysis using Phoenix® WinNonlin® version 8.1. All PK data from the dose escalation/expansion cohorts available at the data cut-off date were reported.

### Endpoints and assessments

Safety parameters included DLTs, AEs, treatment-emergent adverse events (TEAE), and serious adverse events (SAE). A DLT determined to have a reasonable possibility of being related to surufatinib was defined as any grade 4, non-hematological toxicity; any grade 3 non-hematological toxicity except nausea/vomiting, diarrhea, constipation, electrolyte imbalances, or transient hypertension downgraded within 3 days with appropriate supportive treatment; grade 4 neutropenia lasting > 7 days; grade 3 febrile neutropenia (absolute neutrophil count of < 1.0 × 10^9^/L, with a single temperature of > 38.3 °C or a sustained temperature of ≥ 38 °C for > 1 h); grade 4 thrombocytopenia or grade ≥ 3 thrombocytopenia associated with tendency to bleed; >14 day dose interruption or delay due to toxicity; any life-threatening complication or abnormality not covered in the National Cancer Institute Common Terminology Criteria for Adverse Events version 4.03. The MTD was the highest dose reached with no more than 1 DLT among 6 evaluable patients.

The primary efficacy endpoint for the pNET and epNET cohorts in dose expansion was progression-free survival (PFS) rate at 11 months (according to RECIST v1.1) defined as the time from date of first dose until date of objective progression or death (by any cause in the absence of progression). Additional antitumor activity assessments per RECIST v1.1 included objective response rate (ORR) defined as the proportion of patients with a best overall response (BOR) of complete response (CR) or partial response (PR); disease control rate (DCR) defined as the proportion of patients whose BOR from baseline was either a CR, PR, or stable disease (SD); time to response (TTR) defined as the time between date of first dose until first documented response (CR or PR); duration of response (DoR) defined as time from the first time that the objective response reached CR or PR, whichever came first, until the occurrence of progressive disease (PD) or death (if the death occurred before recording the PD); and percent change from baseline of target lesion calculated at each visit. PR or CR required changes in tumor measurements confirmed by repeat assessments performed ≥ 4 weeks after the criteria for response were first met.

### Statistical analysis

The analyses presented here are based on a data cut-off date of 30 June 2020. No formal statistical hypotheses were tested. ORR and DCR were estimated, and 95% exact confidence intervals [CIs] were based on the Clopper-Pearson method. The time to event endpoints, PFS, TTR, and DoR were described using Kaplan-Meier method, including estimated median (in months) with 95% CI, 25th and 75th percentiles.

The Safety Analysis Set consisted of all patients who received at least 1 surufatinib dose. The DLT Evaluable Set consisted of all patients in the Safety Analysis Set who were evaluable for DLTs. The Efficacy Analysis Set consisted of all patients who received ≥ 1 dose of surufatinib and had ≥ 1 postbaseline tumor assessment.

## Results

### Patients

In dose escalation, 35 patients were enrolled into 5 dose cohorts. The baseline characteristics were comparable across dose cohorts (Table 1). The most common malignancies were cholangiocarcinoma (4 patients), ovarian cancer (3 patients), and adenocarcinoma of colon, endometrial adenocarcinoma, ovarian epithelial cancer, and rectal adenocarcinoma (2 patients each) (Supplementary Table S1). All other types of malignancies were reported in 1 patient each. In dose expansion, 16 patients each were enrolled in the pNET and epNET cohorts. The baseline characteristics for the expansion cohorts are shown in Table 1.

**Table 1:** Baseline characteristics of the dose escalation and dose expansion NET cohorts

	Dose escalation					Dose expansion NET cohorts	
	Surufatinib dose						
	50 mg (n = 3)	100 mg (n = 7)	200 mg (n = 3)	300 mg (n = 9)	400 mg (n = 13)	pNET (n = 16)	epNET (n = 16)
**Age (years)**, **Median (min, max)**	48.7 (48, 56)	57.8 (43, 69)	58.1 (56, 69)	63.0 (48, 85)	60.6 (29, 75)	64.4 (39, 72)	62.2 (44, 75)
**Age group, n (%)**							
< 65 years	3 (100.0)	6 (85.7)	2 (66.7)	6 (66.7)	9 (69.2)	9 (56.3)	9 (56.3)
≥ 65 years	0	1 (14.3)	1 (33.3)	3 (33.3)	4 (30.8)	7 (43.8)	7 (43.8)
**Gender, n (%)**							
Male	1 (33.3)	1 (14.3)	0	4 (44.4)	4 (30.8)	11 (68.8)	11 (68.8)
Female	2 (66.7)	6 (85.7)	3 (100.0)	5 (55.6)	9 (69.2)	5 (31.3)	5 (31.3)
**Race, n (%)**							
White	3 (100.0)	7 (100.0)	3 (100.0)	9 (100.0)	12 (92.3)	6 (37.5)	9 (56.3)
Asian	0	0	0	0	0	2 (12.5)	0
Black or African American	0	0	0	0	0	0	4 (25.0)
Other	0	0	0	0	1 (7.7)	0	3 (18.8)
Not reported	0	0	0	0	0	8 (50.0)	0
**Ethnicity, n (%)**							
Hispanic or Latino	0	1 (14.3)	0	0	1 (7.7)	1 (6.3)	4 (25.0)
Not Hispanic or Latino	2 (66.7)	6 (85.7)	2 (66.7)	9 (100.0)	11 (84.6)	7 (43.8)	12 (75.0)
Not reported/missing/unknown	1 (33.3)	0	1 (33.3)	0	1 (7.7)	8 (50.0)	0
**ECOG PS**							
0	1 (33.3)	3 (42.9)	1 (33.3)	5 (55.6)	2 (15.4)	3 (18.8)	8 (50.0)
1	2 (66.7)	4 (57.1)	2 (66.7)	4 (44.4)	11 (84.6)	13 (81.3)	8 (50.0)
**Patients with prior oncology chemotherapy and medication, n (%)**	3 (100.0)	6 ( 85.7)	3 (100.0)	9 (100.0)	13 (100.0)	16 (100.0)	16 (100.0)
Number of prior antitumor systemic therapies, median (min, max)	5.0 (1, 9)	3.0 (1, 10)	5.0 (3, 5)	2.0 (1, 6)	3.0 (1, 9)	4.0 (1, 8)	2.0 (2, 5)
**Patients with prior oncology radiotherapy, n (%)**	1 (33.3)	5 (71.4)	2 (66.7)	5 (55.6)	6 (46.2)	3 (18.8)	8 (50.0)
**Patients with prior oncology surgery and procedures, n (%)**	2 (66.7)	7 (100.0)	3 (100.0)	8 (88.9)	11 (84.6)	12 (75.0)	11 (68.8)

ECOG = Eastern Cooperative Oncology Group; epNET = extrapancreatic neuroendocrine tumor; max = maximum; min = minimum; NET = neuroendocrine tumor; pNET = pancreatic neuroendocrine tumor; PS = performance score

### Pharmacokinetics

Surufatinib plasma concentration data from 35 patients in dose escalation and 72 patients in the 4 cohorts in dose expansion (biliary tract cancer N = 30; pNET N = 16, epNET N = 16; soft tissue sarcoma N = 10) were available for PK analysis. The mean concentration-time profiles of surufatinib on C1D15 by dose are presented in Supplementary Fig. S1; mean PK parameters are shown in Supplementary Table S2.

After a single dose of surufatinib on C1D1, C_max_ values were observed at a median T_max_ of approximately 1 to 4 h. After reaching C_max_, surufatinib concentration declined in a biphasic manner in general.

After daily surufatinib dosing over C1D7 to C1D28, steady-state PK was achieved after the C1D7 dose across all dosing cohorts based on similar median trough concentrations. Median T_max_ for C1D15 and C2D1 across all doses was between 1.9 and 4.0 h. Geometric mean C_max_ and AUC_0 − tau_ values at steady state appeared to increase proportionally with increasing dose. Systemic exposures at steady state (based on C_max_, AUC_0 − tau_, and C_min_) were comparable between C1D15 and C2D1. The accumulation ratio for C1D15 and C2D1 indicated that surufatinib accumulated by approximately 1.5- to 2-fold after repeat dosing.

Dose proportionality was evaluated using a power model that assessed the relationship between surufatinib dose levels and systemic exposure. The 95% CI of the slope estimate for surufatinib AUCs and C_max_ on C1D1, C1D15, and C2D1 included the value of 1, and the slope was contained within 0.8 and 1.2, suggesting dose proportionality for AUCs and C_max_ following surufatinib single- or multiple-dose administration across the 50 to 400 mg dose range (Supplementary Table S3).

### Exposure

For the 35 patients in dose escalation, the median number of cycles of treatment was 3 (min, max: 1, 22), and the median number of days on treatment was 73.0 (28, 604). Median relative dose intensity was 75.4% (37.0, 100.0). Almost all (34 [97.1%]) patients had some dose adjustment or interruption. There were 11 (31.4%) patients with at least 1 dose reduction; of these 1 (2.9%) had ≥ 2 dose reductions. TEAEs leading to dose reduction included fatigue and arthritis (100 mg dose); hypertension (300 mg dose); and alanine aminotransferase (ALT) increase, aspartate aminotransferase (AST) increase, international normalized ratio increase, proteinuria, and nausea (400 mg dose).

For the 16 pNET patients in dose expansion, the median number of cycles of treatment was 8.5 (min, max: 2, 23), and the median number of days on treatment was 244 (62, 618). Median relative dose intensity was 94.4% (46.9, 100.0). There were 14 (87.5%) patients with dose interruption and 6 (37.5%) patients with dose reduction during the study.

For the 16 epNET patients in dose expansion, the median number of cycles of treatment was 8.0 (min, max: 2, 15), and the median number of days on treatment was 214 (29, 394). Median relative dose intensity was 94.2% (64.5, 100.0). There were 11 (68.8%) patients with dose interruption and 4 (25.0%) patients with dose reduction during the study.

### Safety

In dose escalation (n = 35), 32 patients were included in the DLT Evaluable Set. A total of 5 (15.6%) patients reported 6 DLT events within a median of 22 days from the time of first dose to the first DLT event. In this study, the MTD and RP2D were determined to be 300 mg QD based on the occurrence of 3 DLTs reported in 3 separate patients in the 400 mg QD cohort (n = 11). The 3 DLTs, considered related to study drug, consisted of grade 3 platelet count decrease, grade 3 proteinuria, and grade 3 ALT increase. Two DLT events of fatigue (grades 2 and 3) were reported in the same patient in the 100 mg dose cohort. One DLT of grade 4 hypertensive crisis, considered related to study drug, was reported in the 300 mg dose cohort.

**Table 2:** Overview of TEAEs in ≥ 20% of patients in the total treatment group in dose escalation (Safety Analysis Set)

SOC PT n (%)	50 mg (N = 3)	100 mg (N = 7)	200 mg (N = 3)	300 mg (N = 9)	400 mg (N = 13)	Total (N = 35)
**Any TEAE**	3 (100.0)	7 (100.0)	3 (100.0)	9 (100.0)	13 (100.0)	35 (100.0)
**TEAE (grade ≥3)**	1 (33.3)	2 (28.6)	1 (33.3)	6 (66.7)	12 (92.3)	22 (62.9)
**Gastrointestinal disorders**	3 (100.0)	5 (71.4)	3 (100.0)	9 (100.0)	12 (92.3)	32 (91.4)
Diarrhea	1 (33.3)	3 (42.9)	0	2 (22.2)	10 (76.9)	16 (45.7)
Nausea	1 (33.3)	4 (57.1)	1 (33.3)	4 (44.4)	6 (46.2)	16 (45.7)
Vomiting	3 (100.0)	0	1 (33.3)	3 (33.3)	4 (30.8)	11 (31.4)
Abdominal pain	0	2 (28.6)	1 (33.3)	2 (22.2)	2 (15.4)	7 (20.0)
**General disorders and administration site conditions**	1 (33.3)	4 (57.1)	1 (33.3)	7 (77.8)	10 ( 76.9)	23 (65.7)
Fatigue	1 (33.3)	4 (57.1)	0	2 (22.2)	7 (53.8)	14 (40.0)
**Nervous system disorders**	3 (100.0)	3 (42.9)	2 (66.7)	4 (44.4)	5 (38.5)	17 (48.6)
Headache	2 (66.7)	0	1 (33.3)	2 (22.2)	3 (23.1)	8 (22.9)
**Metabolism and nutrition disorders**	1 (33.3)	3 (42.9)	0	3 (33.3)	8 (61.5)	15 (42.9)
Dehydration	0	2 (28.6)	0	3 (33.3)	2 (15.4)	7 (20.0)
**Vascular disorders**	1 (33.3)	0	0	3 (33.3)	5 (38.5)	9 (25.7)
Hypertension	1 (33.3)	0	0	3 (33.3)	4 (30.8)	8 (22.9)

PT = preferred term; SOC = system organ class; TEAE = treatment-emergent adverse event

All 35 patients in dose escalation reported ≥ 1 TEAE (Table 2). In general, TEAEs having higher severity (grade ≥ 3) were more likely to be reported in patients receiving higher doses (300 and 400 mg). The most frequent TEAEs in the total population were nausea and diarrhea in 16 (45.7%) patients each, grade ≥ 3 in 1 (2.9%) patient each; fatigue in 14 (40.0%) patients, grade ≥ 3 in 2 (5.7%) patients; vomiting in 11 (31.4%) patients, no grade ≥ 3 events; headache in 8 (22.9%) patients, no grade ≥ 3 events; and hypertension in 8 (22.9%) patients, grade ≥ 3 in 4 (11.4%) patients (Table 2). All other TEAEs were reported in ≤ 20% of patients in the total treatment group. Approximately two-thirds of the total treatment group reported severe TEAEs, with more patients in the higher dose cohorts reporting severe TEAEs. The most frequent severe TEAE was hypertension, in 4 (11.4%) patients in the higher dose cohorts (300 and 400 mg).

SAEs occurred in a third of the dose escalation treatment cohort. SAEs reported in > 1 patient in the total safety population were pneumonia, pleural effusion, and disease progression; none of which was reported in the same dose cohort.

Two patients died during dose escalation due to disease progression; 1 patient in the 100 mg cohort and 1 patient in the 300 mg cohort. Four patients reported TEAEs leading to study drug discontinuation during dose escalation: 1 due to hypertensive crisis in the 300 mg dose cohort; and 1 each due to hypertension, platelet count decrease, and posterior reversible encephalopathy syndrome in the 400 mg dose cohort (Supplementary Table S4).

More than half of the patients in dose escalation reported treatment-emergent adverse events of special interest (AESI). AESIs of scientific and medical interest for surufatinib included hepatic disorder, proteinuria, hypertension, thyroid dysfunction, hemorrhage, and acute renal failure. AESIs were reported by more patients in higher dose cohorts (300 and 400 mg) than lower dose cohorts. The most commonly reported AESI categories were hepatic disorders and hypertension, each reported in 8 (22.9%) patients.

**Table 3:** TEAEs in > 20% of patients in the pNET or epNET Cohort in dose expansion (safety analysis set)

	pNET (N = 16)		epNET (N = 16)	
SOC PT n (%)	Any grade	Grade ≥ 3	Any grade	Grade ≥ 3
**Any TEAE**	16 (100.0)	11 (68.8)	16 (100.0)	13 (81.3)
**Gastrointestinal disorders**	12 (75.0)	-	12 (75.0)	-
Abdominal pain	6 (37.5)	0	4 (25.0)	0
Diarrhea	5 (31.3)	1 (6.3)	6 (37.5)	2 (12.5)
Nausea	3 (18.8)	1 (6.3)	5 (31.3)	0
Vomiting	4 (25.0)	1 (6.3)	5 (31.3)	0
Decreased appetite	2 (12.5)	0	4 (25.0)	0
Constipation	2 (12.5)	0	4 (25.0)	0
Abdominal distension	4 (25.0)	0	2 (12.5)	0
**General disorders and administration site conditions**	10 (62.5)	-	12 (75.0)	-
Fatigue	4 (25.0)	0	11 (68.8)	1 (6.3)
Edema peripheral	5 (31.3)	0	2 (12.5)	1 (6.3)
Rash	4 (25.0)	0	0	0
**Renal and urinary disorders**	10 (62.5)	-	9 (56.3)	-
Proteinuria	7 (43.8)	1 (6.3)	5 (31.3)	1 (6.3)
**Nervous system disorders**	6 (37.5)	-	6 (37.5)	-
Headache	4 (25.0)	0	3 (18.8)	1 (6.3)
**Metabolism and nutrition disorders**	11 (68.8)	-	6 (37.5)	-
**Vascular disorders**	7 (43.8)	-	8 (50.0)	-
Hypertension	7 (43.8)	6 (37.5)	7 (43.8)	6 (37.5)

epNET = extrapancreatic neuroendocrine tumor; pNET = pancreatic neuroendocrine tumor; PT = preferred term; SOC = system organ class; TEAE = treatment-emergent adverse event

There were no clinically meaningful trends in mean changes from baseline for any vital sign variable or clinical laboratory variable. No clinically relevant changes in QT interval corrected by Fridericia’s formula were observed in patients treated with 50 to 400 mg QD surufatinib.

All 16 patients in the pNET and epNET cohorts were included in the Safety Analysis Set and reported ≥ 1 TEAE. The most frequent TEAEs in the NET cohorts were fatigue, hypertension, proteinuria, and diarrhea (Table 3). A higher percentage of patients in the epNET cohort (68.8%) experienced fatigue compared to the pNET cohort (25.0%). Eleven (68.8%) patients in the pNET cohort and 13 (81.3%) patients in the epNET cohort reported a TEAE with grade ≥ 3 severity; the most common in both cohorts was hypertension.

In the pNET cohort, 13 (81.3%) patients reported a drug related TEAE; the most common were hypertension (43.8%) and proteinuria (31.3%). In the epNET cohort, 15 (93.8%) patients reported a drug related TEAE; the most common were fatigue (50.0%), hypertension (43.8%), and diarrhea (31.3%). One patient in the pNET cohort reported a serious TEAE of sepsis, and 1 patient in the epNET cohort reported a serious TEAE of arthralgia. No patients in the NET cohorts died during dose expansion. Two (12.5%) patients in the epNET cohort reported a TEAE of proteinuria leading to drug discontinuation (Supplementary Table S4).

There were no clinically meaningful trends in mean changes from baseline for any clinical laboratory variable, including hematology, serum chemistry, coagulation, thyroid function, and urinalysis in dose expansion. One patient in the pNET cohort and 2 patients in the epNET cohort had an AST/ALT ratio > 3 times and ≤ 5 times the upper limit of normal.

**Table 4:** PFS, BOR, ORR, DCR, and TTR by disease cohort in dose expansion

	pNET (N = 16)	epNET (N = 16)	Total (N = 32)
**Number of patients with PFS events (%)** ^**a**^	8 (50.0)	5 (31.3)	13 (40.6)
**Median (95% CI)**	15.2 (5.2, NE)	11.5 (6.5, 11.5)	11.5 (6.5, 17.5)
PFS rate (%) at 4 months (95% CI)	93.3 (61.3, 99.0)	93.8 (63.2, 99.1)	93.4 (76.2, 98.3)
PFS rate (%) at 11 months (95% CI)	57.4 (28.7, 78.2)	51.1 (12.8, 80.3)	55.6 (32.3, 73.7)
**BOR, n (%)**			
CR	0	0	0
PR	3 (18.8)	1 (6.3)	4 (12.5)
SD^b^	11 (68.8)	14 (87.5)	25 (78.1)
PD	1 (6.3)	1 (6.3)	2 (6.3)
NE^c^	1 (6.3)	0	1 (3.1)
**Confirmed ORR (CR + PR), n (%)**	3 (18.8)	1 (6.3)	4 (12.5)
**DCR (CR + PR + SD), n (%)**	14 (87.5)	15 (93.8)	29 (90.6)
**TTR**			
Number of responders	3	1	4
TTR months, median (95% CI)	2.8 (2.7, 4.6)	2.5 (NE, NE)	2.7 (2.5, 4.6)

BOR = best overall response; CI = confidence interval; CR = complete response; DCR = disease control rate; epNET = extrapancreatic neuroendocrine tumor; max = maximum; min = minimum; NE = not evaluable; ORR = objective response rate; PD = progressive disease; PFS = progression-free survival; pNET = pancreatic neuroendocrine tumor; PR = partial response; SD = stable disease; TTR = time to response

^a^ All patients were included; patients without PFS event were censored.

^b^ SD after at least 8 weeks from start of treatment

^c^ SD too short; <53 days.

Notes: The tumor response was determined according to the international Response Evaluation Criteria in Solid Tumors guideline version 1.1. The median estimates and percentiles were based on the Kaplan-Meier approach. ORR was defined as the proportion of patients achieving a CR or PR as confirmed BOR. DCR was defined as the proportion of patients achieving a CR, PR, or SD as confirmed BOR. The denominator for the calculation of ORR and DCR was all patients in the Efficacy Analysis Set. The 95% 2-sided exact CIs of ORR and DCR were based on the Clopper-Pearson method. The TTR was defined as the time between the start date of study drug until the first documented response according to Response Evaluation Criteria in Solid Tumors version 1.1, and the percentages were calculated based on the number of patients with objective response (complete response or PR). The median estimates and percentiles and the estimated TTR were based on the Kaplan-Meier approach.

### Antitumor efficacy

In dose escalation (n = 35), 31 patients were evaluable for tumor response by RECIST v1.1. No patient achieved a CR or PR. A total of 17 (54.8%) patients had a BOR of SD. The overall DCR (CR + PR + SD) was 54.8% (17 patients); in the 300 mg dose cohort, the DCR was 50% (4 of 8 patients).

**Fig. 1:**
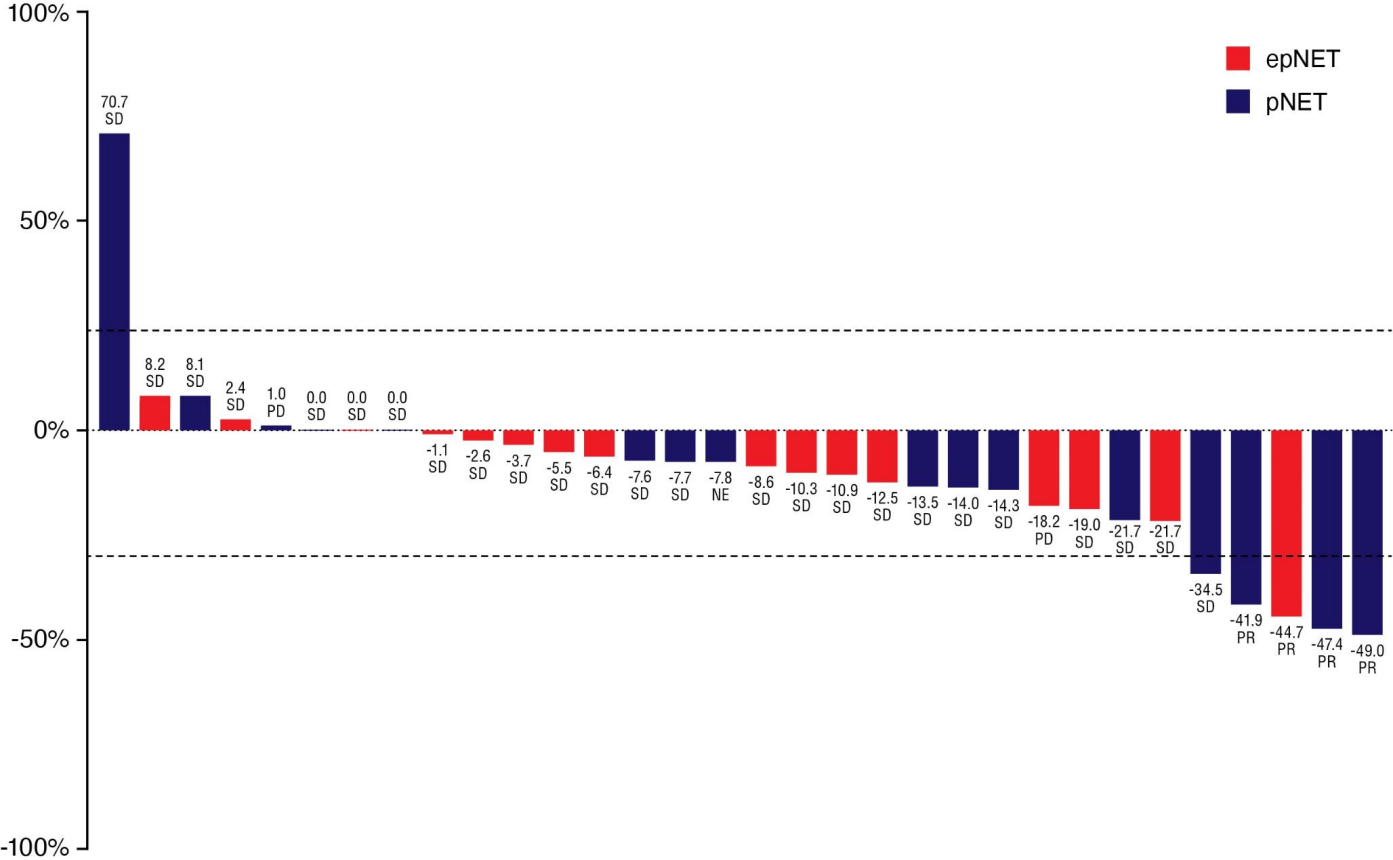
***Best percentage change of target lesion diameter in the NET cohorts*** Abbreviations: epNET = extrapancreatic neuroendocrine tumor; NET = neuroendocrine tumor; PD = progressive disease; pNET = pancreatic neuroendocrine tumor; PR = partial response; SD = stable disease

The primary efficacy endpoint for dose expansion was Investigator-assessed PFS (Supplementary Fig. S2). In the pNET cohort (n = 16), there were 8 (50.0%) PFS events, and the median PFS was 15.2 months (95% CI: 5.2, NE). In the epNET cohort (n = 16), there were 5 (31.3%) PFS events, and the median PFS was 11.5 months (95% CI: 6.5, 11.5). The estimated PFS rate at 4 months was 93.3% (95% CI: 61.3, 99.0) and 93.8% (95% CI: 63.2, 99.1) for the pNET and epNET cohort, respectively. The estimated PFS rate at 11 months was 57.4% (95% CI: 28.7, 78.2) and 51.1% (95% CI: 12.8, 80.3) for the pNET and epNET cohort, respectively. No patients achieved a CR (Table 4). Three (18.8%) patients and 1 (6.3%) patient in the pNET and epNET cohort, respectively, achieved a confirmed PR. The DCR estimate (CR + PR + SD) was 87.5% (14 patients) and 93.8% (15 patients) in the pNET and epNET cohort, respectively. The median TTR for the 3 patients with a PR in the pNET cohort was 2.8 months (95% CI: 2.7, 4.6), and for the 1 patient in the epNET cohort, TTR was 2.5 months. Median DoR had not been reached at the time of interim analysis. (Table 4). The best percentage change from baseline in the sum of target lesion diameters is illustrated for the pNET and epNET cohorts in Fig. 1.

## Discussion

In this study, the RP2D of surufatinib in US patients was 300 mg QD, consistent with the RP2D determined in clinical studies performed in China [10]. The PK results for surufatinib in this US study were comparable to those observed in the previous Phase 1b/2 study in patients with NETs in China (NCT02267967) [11]. In the study conducted in China (N = 81), surufatinib C_max_ and AUC_0 − tau_ following 300 mg QD on C1D14 were 487 ng/mL and 4810 ng*h/mL, respectively; compared to 456 ng/mL and 4770 ng*h/mL, respectively on C1D15 in the US population. These results suggest that the PK of surufatinib in US patients is similar to that seen in Chinese patients and therefore, no clinically meaningful PK difference exists between patients from China and the US.

Surufatinib was generally well tolerated at 300 mg QD in US patients consistent with studies conducted in China; the TEAEs were manageable and resolved after withdrawal of surufatinib. The most common TEAEs in this study were hypertension and proteinuria, consistent with earlier surufatinib studies conducted in China [8, 9] and consistent with the AEs associated with angiogenesis inhibitors [12].

The primary objective of dose expansion was to evaluate the antitumor activity of surufatinib at the RP2D evaluated by PFS rate at 11 months. For the pNET cohort, estimated PFS rate at 11 months was 57.4% (95% CI: 28.7, 78.2) and 51.1% (95% CI: 12.8, 80.3) for the epNET cohort. Overall, 6 patients (3 pNET and 3 epNET) received concomitant SSA for control of symptoms related to functional tumors, and 3 of these patients achieved a PR despite prior disease progression on SSAs.

Despite a heavily pretreated refractory patient population, the antitumor activity observed in dose expansion is broadly consistent with 2 completed, Phase 3, randomized, double-blind, placebo-controlled studies in patients with advanced pNETs (NCT02589821) [8] and advanced epNETs (NCT02588170) [9], conducted in China. The patient populations in the studies conducted in China received up to 2 prior types of systemic therapy, including cytotoxic chemotherapeutics (most common prior therapy), SSAs, and targeted kinase inhibitors. Therefore, the results from the studies conducted in China could potentially inform treatment decisions for patients with advanced NETs outside of China.

These clinical findings in US patients support the applicability of earlier surufatinib studies in this patient population and further establish the safety and antitumor efficacy of 300 mg QD oral surufatinib monotherapy for the treatment of pNETs and epNETs. Investigation of surufatinib in NETs and other solid tumor types is ongoing globally, including combination trials with tislelizumab, an anti-programmed cell death 1 antibody, and gemcitabine.

## Electronic supplementary material

Below is the link to the electronic supplementary material.

Supplementary Material 1

## Data Availability

Data generated or analyzed during this study are included in this published article and its supplementary information files. Additional data/analyses can be provided upon request.
