# Supplementary material for: A dose escalation/expansion study evaluating dose, safety, and efficacy of the novel tyrosine kinase inhibitor surufatinib, which inhibits VEGFR 1, 2, & 3, FGFR 1, and CSF1R, in US patients with neuroendocrine tumors
Source: Invest New Drugs. 2023 Apr 19;41(3):421–30. doi: 10.1007/s10637-023-01359-2 (PMC10289989; doi:10.1007/s10637-023-01359-2)
Supplement: Supplementary file 1 — Supplementary Material 1 [file 10637_2023_1359_MOESM1_ESM.pdf]

Supplementary Information

A dose escalation/expansion study evaluating dose, safety, and efficacy of the novel tyrosine kinase inhibitor surufatinib, which inhibits VEGFR 1, 2, & 3, FGFR 1, and CSF1R, in US patients with neuroendocrine tumors

Arvind Dasari[1], Erika P. Hamilton[2], Gerald S. Falchook[3], Judy S. Wang[4], Daneng Li[5], Max W. Sung[6], Caly Chien[7], Shivani Nanda[7], Christopher Tucci[7], Marjo Hahka-Kemppinen[7], Andrew Scott Paulson[8]

[1]MD Anderson Cancer Center, Houston, TX, USA; [2]Sarah Cannon Research Institute/Tennessee Oncology, Nashville, TN, USA; [3]Sarah Cannon Research Institute at HealthONE, Denver, CO, USA; [4] Florida Cancer Specialists/Sarah Cannon Research Institute, Sarasota, FL, USA, [5]City of Hope Comprehensive Cancer Center and Beckman Research Institute, Duarte, CA, USA; [6]Tisch Institute at The Icahn School of Medicine at Mount Sinai, New York, NY, USA; [7]HUTCHMED International Corporation, Florham Park, NJ, USA; [8]Baylor Sammons Cancer Center, Dallas, TX, USA

Supplementary Table S1. Primary Tumor Site

Primary Tumor site, n (%)	Dose escalation Surufatinib dose						Dose expansion Disease cohort	
	**50 mg (n=3)**	**100 mg (n=7)**	**200 mg (n=3)**	**300 mg (n=9)**	**400 mg (n=13)**	**Total (n=35)**	**pNET (n=16)**	**epNET (n=16)**
Adenocarcinoma of colon	0	0	0	0	2 (15.4)	2 (5.7)	0	0
Adenocarcinoma pancreas	0	0	0	1 (11.1)	0	1 (2.9)	0	0
Breast cancer metastatic	0	0	1 (33.3)	0	0	1 (2.9)	0	0
Carcinoid tumor	0	0	0	0	0	0	0	4 (25.0)
Carcinoid tumor of the gastrointestinal tract	0	0	0	0	0	0	0	1 (6.3)
Cervix carcinoma	0	0	0	0	1 (7.7)	1 (2.9)	0	0
Cholangiocarcinoma	0	0	1 (33.3)	1 (11.1)	2 (15.4)	4 (11.4)	0	0
Chondrosarcoma	0	1 (14.3)	0	0	0	1 (2.9)	0	0
Endometrial adenocarcinoma	0	1 (14.3)	0	0	1 (7.7)	2 (5.7)	0	0
Endometrial cancer	0	1 (14.3)	0	0	0	1 (2.9)	0	0
Endometrial neoplasm	0	0	0	0	1 (7.7)	1 (2.9)	0	0
Fallopian tube cancer	0	0	0	0	1 (7.7)	1 (2.9)	0	0
Fibrosarcoma	0	0	0	0	1 (7.7)	1 (2.9)	0	0
Follicular thyroid cancer	0	0	0	1 (11.1)	0	1 (2.9)	0	0
Hemangiopericytoma	0	0	0	1 (11.1)	0	1 (2.9)	0	0
Intestinal adenocarcinoma	1 (33.3)	0	0	0	0	1 (2.9)	0	0
Invasive breast carcinoma	0	1 (14.3)	0	0	0	1 (2.9)	0	0
Invasive ductal breast carcinoma	1 (33.3)	0	0	0	0	1 (2.9)	0	0
Leiomyosarcoma	0	0	0	0	1 (7.7)	1 (2.9)	0	0
Malignant neoplasm of unknown primary site	0	0	0	0	1 (7.7)	1 (2.9)	0	0
Metastatic carcinoid tumor	0	0	0	0	0	0	0	1 (6.3)
Neuroendocrine carcinoma	0	0	0	0	1 (7.7)	1 (2.9)	3 (18.8)	9 (56.3)
Non-small cell lung cancer	0	0	0	0	0	0	0	1 (6.3)
Ovarian cancer	0	0	0	3 (33.3)	0	3 (8.6)	0	0

Primary Tumor site, n (%)	Dose escalation Surufatinib dose						Dose expansion Disease cohort	
	50 mg (n=3)	100 mg (n=7)	200 mg (n=3)	300 mg (n=9)	400 mg (n=13)	Total (n=35)	pNET (n=16)	epNET (n=16)
Ovarian epithelial cancer	0	1 (14.3)	1 (33.3)	0	0	2 (5.7)	0	0
Pancreatic carcinoma	0	0	0	0	1 (7.7)	1 (2.9)	0	0
Pancreatic neuroendocrine tumor	1 (33.3)	0	0	0	0	1 (2.9)	12 (75.0)	0
Pancreatic neuroendocrine tumor metastatic	0	0	0	0	0	0	1 (6.3)	0
Paraganglion neoplasm	0	0	0	1 (11.1)	0	1 (2.9)	0	0
Peritoneal carcinoma metastatic	0	1 (14.3)	0	0	0	1 (2.9)	0	0
Rectal adenocarcinoma	0	1 (14.3)	0	1 (11.1)	0	2 (5.7)	0	0

epNET=extrapancreatic neuroendocrine tumor; pNET=pancreatic neuroendocrine tumor

Supplementary Table S2. Mean Pharmacokinetic parameters of surufatinib

Visit	N	Dose (mg)	T_{max} (h)	C_{max} (ng/mL)	AUC_{0-24} or AUC_{0-tau} (h*ng/mL)	C_{min} (ng/mL)	CL_{ss}/F (L/h)	AR_AUC_{0-tau}
C1D1	3	50	2.00 (1.02-2.17)	73.6 (39.2)	616 (7.5)	---	---	---
	7	100	1.03 (1.00-4.00)	149 (107.3)	877 (81.2)	---	---	---
	3	200	2.05 (1.02-3.58)	180 (39.3)	1360 (10.5)	---	---	---
	79	300	2.20 (0.75-23.7)	364 (66.6)	3080 (58.8)[a]	---	---	---
	13	400	3.83 (1.18-8.13)	427 (82.9)	3540 (76.8)[b]	---	---	---
C1D15	3	50	3.98 (1.97-4.00)	99.0 (16.9)	968 (1.5)	17.4 (8.9)	51.7 (1.5)	1.57 (6.1)
	7	100	2.00 (0.98-3.88)	167 (109.0)	1320 (95.2)	22.0 (80.8)	75.6 (95.2)	1.51 (38.6)
	3	200	2.03 (1.00-2.12)	261 (43.6)	2310 (36.7)	40.8 (29.5)	86.7 (36.7)	1.69 (34.4)
	70	300	3.50 (0.73-7.88)	456 (68.0)	4770 (64.4)[c]	73.3 (150.6)	62.9 (64.4)[c]	1.62 (38.6)[d]
	11	400	4.00(2.00-7.07)	566 (71.0)	6890 (60.5)[e]	131 (68.5)	58.1 (60.5)[e]	2.02 (40.5)[f]
C2D1	3	50	2.00 (1.05-2.02)	121 (11.4)	1170 (16.5)	24.9 (13.6)	42.8 (16.5)	1.90 (19.9)
	5	100	1.93 (0.93-2.07)	162 (143.0)	1340 (94.2)	22.4 (77.7)	74.9 (94.2)	1.60 (41.5)
	1	200	2.03	411	3470	56.3	57.6	---
	22	300	3.76 (0.95-7.38)	465 (53.6)	4860 (44.4)[g]	86 (54.2)	61.8 (44.4)[g]	1.66 (38.6)[h]
	7	400	3.78 (1.88-4.20)	631 (71.0)	7720 (66.5)	147 (54.7)	51.8 (66.5)	1.59 (34.1)[i]

AR_AUC_{0-tau}=accumulation ratio for area under the plasma concentration versus time curve over the dosing interval; AUC_{0-24}=area under the plasma concentration-time curve from time 0 to 24 hours; AUC_{0-tau}=area under the plasma concentration versus time curve over the dosing interval; C=cycle; CL_{ss}/F= apparent clearance at steady state; C_{max}=maximum plasma concentration; C_{min}=minimum plasma concentration at steady state; CV%=coefficient of variation; D=day; max=maximum; min=minimum; T_{max}=time to maximum plasma concentration

[a]N=68; [b]N=12; [c]N=67; [d]N=58; [e]N=10; [f]N=9; [g]N=18; [h]N=17; [i]N=6

Notes: T_{max} is presented as median (min-max). C_{max}, AUC_{0-24}, AUC_{0-tau}, CL_{ss}/F, and AR_AUC_{0-tau} are presented as geometric mean (geometric CV%). AUC_{0-24} for C1D1; AUC_{0-tau} for C1D15 and C2D1. Individual values were reported when N=1.

Supplementary Table S3. Dose proportionality evaluation of surufatinib after oral administration

Visit	Dependent	Slope	Standard error	Denom_DF	T_critical	95% CI (lower)	95% CI (upper)
C1D1	LnC_{max}	0.849	0.145	103	1.98	0.560	1.14
C1D1	$LnAUC_{0-24}$	0.989	0.132	91	1.99	0.727	1.25
C1D15	LnC_{max}	0.882	0.146	92	1.99	0.593	1.17
C1D15	$LnAUC_{0-tau}$	1.05	0.139	88	1.99	0.772	1.32
C2D1	LnC_{max}	0.843	0.156	36	2.03	0.525	1.16
C2D1	$LnAUC_{0-tau}$	0.986	0.139	32	2.04	0.703	1.27

AUC_{0-24}=area under the plasma concentration-time curve from time 0 to 24 hours; AUC_{0-tau}=area under the plasma concentration versus time curve over the dosing interval; C=cycle; CI=confidence interval; C_{max}=maximum observed plasma concentration; D=day; Denom_DF=denominator degrees of freedom; Ln=natural logarithmic transformation.

Notes: T_critical=t(1-α),df (where α is the statistical significance level, df is degrees of freedom)

Supplementary Table S4. Overview of TEAEs (Dose Escalation and Dose Expansion)

	**Dose escalation, n (%)**						**Dose expansion, n (%)**	
	**50 mg (N=3)**	**100 mg (N=7)**	**200 mg (N=3)**	**300 mg (N=9)**	**400 mg (N=13)**	**Total (N=35)**	**pNET (N=16)**	**epNET (N=16)**
Any TEAE	3 (100.0)	7 (100.0)	3 (100.0)	9 (100.0)	13 (100.0)	35 (100.0)	16 (100.0)	16 (100.0)
TEAE (grade ≥3)	1 (33.3)	2 (28.6)	1 (33.3)	6 (66.7)	12 (92.3)	22 (62.9)	11 (68.8)	13 (81.3)
Any TEAE leading to drug discontinuation	0	0	0	1 (11.1)	3 (23.1)	4 (11.4)	2 (12.5)	5 (31.3)
Any TEAE leading to dose reduction	0	1 (14.3)	0	2 (22.2)	7 (53.8)	10 (28.6)	5 (31.3)	4 (25.0)
Any TEAE leading to drug interruption	0	3 (42.9)	0	4 (44.4)	9 (69.2)	16 (45.7	8 (50.0)	10 (62.5)
Any TEAE leading to death	0	1 (14.3)	0	1 (11.1)	0	2 (5.7)	0	0

epNET=extrapancreatic neuroendocrine tumor; pNET=pancreatic neuroendocrine tumor; TEAE=treatment-emergent adverse event

Notes: The percentages were calculated based on the Safety Analysis Set. TEAEs were defined as any adverse events that started or worsened in severity on or after the first administration date of study drug and no later than 30 (+7) days after the last administration date of study drug or initiation of new antitumor therapy (whichever occurred first). An exception was that study drug related serious adverse events collected later than 37 days after the last dosing date were treated as TEAEs. Adverse event severities were graded according to the National Cancer Institute Common Terminology Criteria for Adverse Events version 4.03. For severity and causal relationship summaries, in the case that a patient reported multiple TEAEs, the TEAE with worst severity and strongest relationship was used in the corresponding summaries. For all other summaries, patients with multiple events were counted for all applicable categories.

Supplementary Figure S1. Mean (StDev) concentration-time profiles of surufatinib in plasma (log-linear) on C1D15 following 50 to 400 mg QD dosing

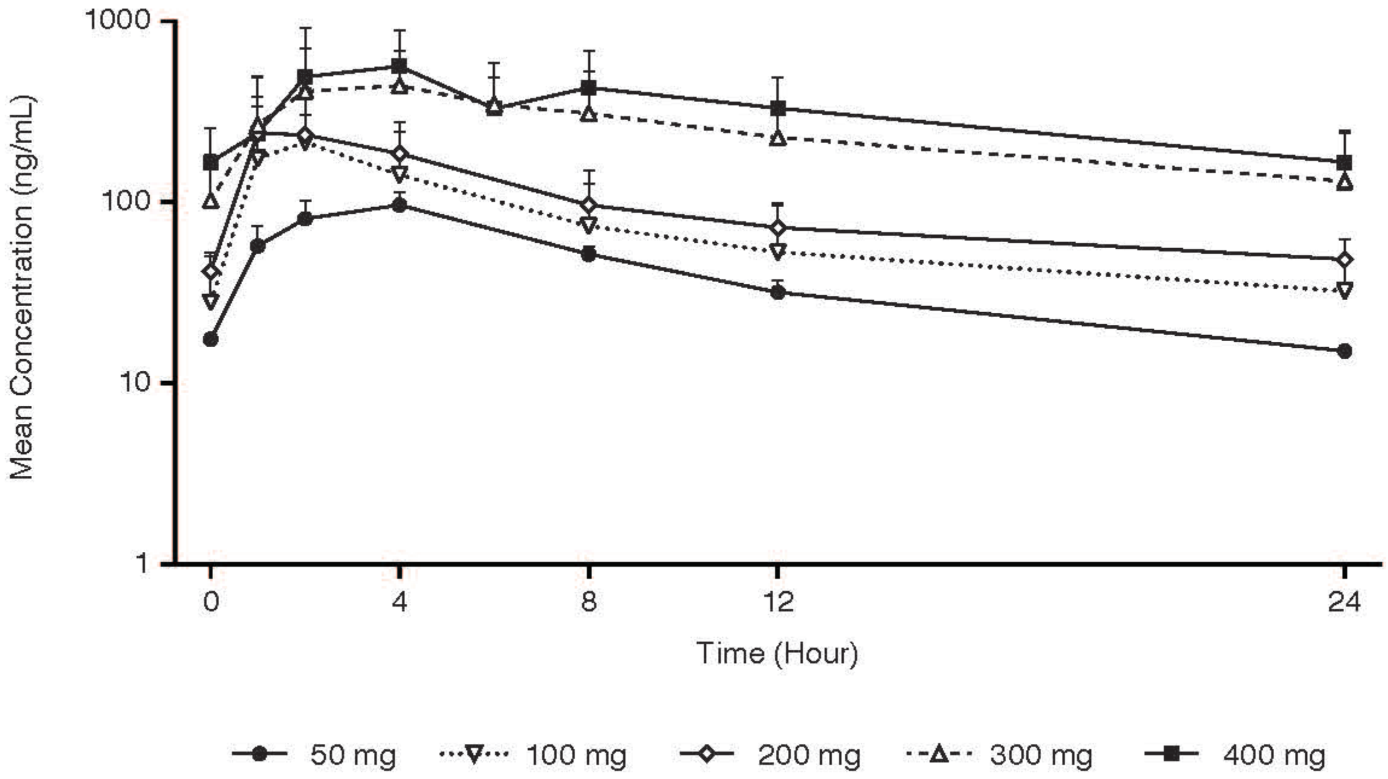

C=cycle; D=day; QD=once daily; StDev=standard deviation

Supplementary Figure S2. Kaplan-Meier curve of progression-free survival (Dose Expansion)

pNET Cohort

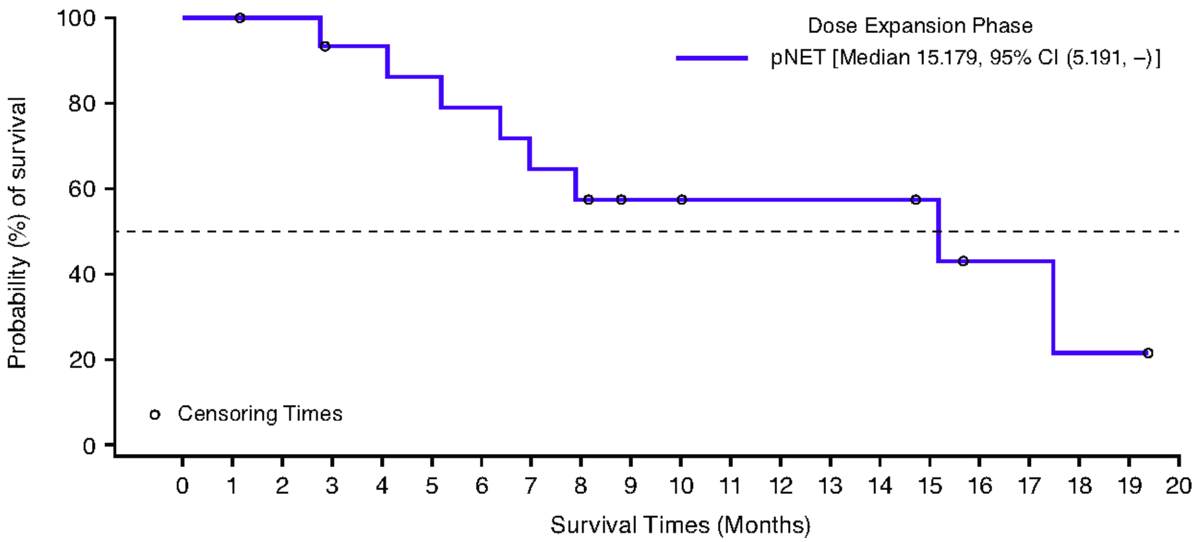

Number of subjects at risk

pNET	16	16	15	13	13	12	11	9	8	6	6	5	5	5	5	4	2	2	1	1	0

epNET Cohort

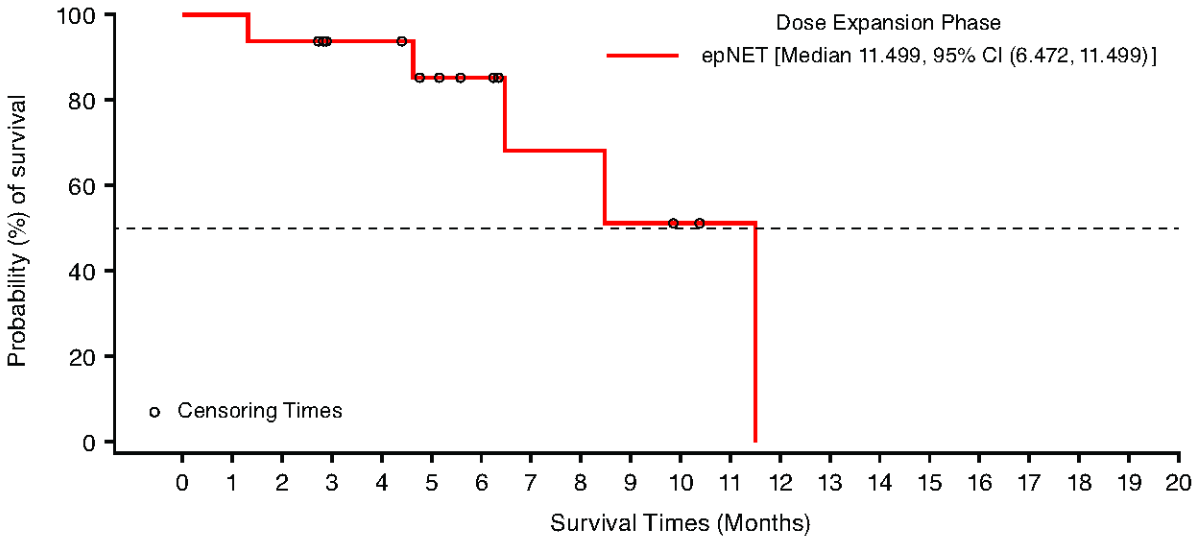

Number of subjects at risk

epNET	16	16	15	12	12	9	7	4	4	3	2	1	0

CI=confidence interval; epNET=extrapancreatic neuroendocrine tumor; pNET=pancreatic neuroendocrine tumor
